# An Overview of Kinematic and Calibration Models Using Internal/External Sensors or Constraints to Improve the Behavior of Spatial Parallel Mechanisms

**DOI:** 10.3390/s101110256

**Published:** 2010-11-16

**Authors:** Ana C. Majarena, Jorge Santolaria, David Samper, Juan J. Aguilar

**Affiliations:** Department of Design and Manufacturing Engineering, Centro Politécnico Superior, University of Zaragoza, c/ María de Luna, 3, 50018 Zaragoza, Spain; E-Mails: jsmazo@unizar.es (J.S.); dsamper@unizar.es (D.S.); jaguilar@unizar.es (J.J.A.)

**Keywords:** parallel mechanism, kinematic, internal sensor calibration, external sensor calibration, constraint calibration

## Abstract

This paper presents an overview of the literature on kinematic and calibration models of parallel mechanisms, the influence of sensors in the mechanism accuracy and parallel mechanisms used as sensors. The most relevant classifications to obtain and solve kinematic models and to identify geometric and non-geometric parameters in the calibration of parallel robots are discussed, examining the advantages and disadvantages of each method, presenting new trends and identifying unsolved problems. This overview tries to answer and show the solutions developed by the most up-to-date research to some of the most frequent questions that appear in the modelling of a parallel mechanism, such as how to measure, the number of sensors and necessary configurations, the type and influence of errors or the number of necessary parameters.

## 1. Introduction

In recent years a number of specialized papers presenting the most relevant methods for modelling and calibrating serial robots have been published, but these methods are not always suitable for parallel robots. With this in mind, this paper is intended to be a guide for those researchers that are familiar with designing, modelling and calibrating parallel mechanisms.

Parallel kinematic systems are closed-chain mechanisms in which the moving platform is joined to the base by two or more independent kinematic chains. In recent years these mechanisms have developed extensively [1] because of their advantages in terms of high loads, stiffness, speed and low moving inertia [1,2]. However, it is difficult to obtain [3–5] the forward kinematic model and the analysis of the singularities of the system [6]. The workspace is limited and is difficult to calculate [1,7].

One of the first moving platforms was patented by Gwinnett in 1931 for the entertainment industry [8], and in 1942 Pollard developed a five Degrees-Of-Freedom (5-DOF) robot for a spray painting machine, featuring three kinematic chains with universal joints [9]. In 1947, Gough designed a parallel robot with six actuators to test tyres. This system had an octahedral hexapod structure [10]. In 1965, Stewart presented a 6-DOF platform for a flight simulator, based on Gough’s platform. This mechanism consisted of six linear actuators joined to the base by universal or spherical ball joints and to the moving platform by spherical ball joints—this publication represented the beginning of parallel robot development. Hunt pointed out the advantages of parallel mechanisms and in 1993 designed a new 6-DOF prototype with rotary actuators [11]. A number of systems have been developed based on this design, such as the manipulator presented by Clavel [12] for pick-and-place operations.

On the basis of these designs, in recent years new families of manipulators have been developed with improved performances, obtaining multiple mechanisms of 3-, 4- and 6-DOF. However, few researchers have developed 2- and 5-DOF systems due to the fact that constraints have to be added for these two types of systems.

Once the design is developed, a calibration process must be carried out in order to evaluate and improve the accuracy of the moving platform. The first decision that the researcher has to make in the calibration process is the calibration method. Once the calibration model is defined, the number and type of internal or external sensors required, the data acquisition procedure, the necessary configurations and the calibration model are determined. The model will determine the nature and number of necessary parameters. Finally, the evaluation of the results and the correction model will allow the accuracy of the system to be improved.

The following sections present examples of manipulators with different number of DOFs, focusing mainly on prototypes developed in the last ten years. A summary of the most relevant methods is presented, with respect to the kinematic model and the calibration of parallel robots, and also new trends such as the methods based on matrix computation or computational intelligence.

## 2. Parallel Robot Design

A classification of the most well-known parallel robots depending on their number of DOF can be found in [13,14]. This section shows some examples of parallel robots, classified by the number of DOF, focusing mainly on the new prototypes developed in the last ten years. Most of them are based on the Stewart platform. Table 1 presents the symbols used for denominating the different joints:

### 2.1. Two-DOF Designs

There are little literature focusing on 2-DOF spatial parallel mechanisms. An analysis of different alternatives of 2-DOF rotational parallel systems can be found in [15]. The kinematic chains, joined to the fixed and moving platforms, result in mechanisms with from 3- to 6-DOF. To reduce the number of DOF to 2, constraints must be added.

**Figure 1.**
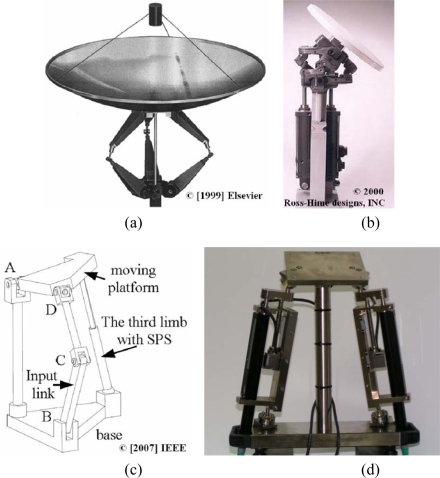
Two-DOF parallel robots: **(a)** Dunlop design (reprinted from [16] with permission from Elsevier). **(b)** Omni-wrist from Rosheim [17] (reprinted with permission from Rosheim). **(c)** Zeng design (reprinted from [15] with permission from IEEE). **(d)** Majarena design [18].

Figure 1(a) shows the Canterbury Tracker by Dunlop [16], an antenna tracking mechanism, in which two kinematic chains are joined to the platform by revolute joints and the third chain is connected via spherical joints, thereby obtaining the azimuth and elevation movements. The Omni-Wrist of Rosheim [Figure 1(b)] in [17], is a new 2-DOF system in which two actuators connecting two plates control a passive plate. This system is able to turn 90 degrees without singularities and it is used in applications such as positioning antennas or robotic surgery. Zeng [15] developed a family of 2-DOF rotational parallel mechanisms and analyzed their mobility. All these systems have a four-bar linkage and contain a moving platform and a fixed base connected by three limbs. An example of parallel mechanism is presented in Figure 1(c). This is a system with three chains U-PRU-SPS where the motion of the moving platform is decoupled. Computation is simple and the system allows us to perform a fast real-time control.

In [18], Majarena designed a pan-tilt parallel platform for the positioning and orientation of two high-precision cameras. This mechanism has two actuators and two linear optical sensors joined to the base and to the platform by high-precision spherical ball joints. The mechanism incorporates a third chain, which is fixed to the base and joined to the platform by a universal joint to obtain the two rotations desired [see Figure 1(d)]. The sensor accuracy is ±1 μm. Given a measurement field of the 2-DOF structure and fixed sensors, a method to use these sensors to increase the angle resolution within this measurement field is presented. The aim of the study is to develop a methodology for improving the mechanism design. The algorithm obtains the anchorage spherical ball joint points and the inclination of the spherical ball supports to increase movement resolution, assuming a fixed feedback resolution on the linear sensor, and, at the same time, decreasing the dimensions of the system and allowing the desired workspace to be obtained. This work develops the kinematic model of a 2-DOF parallel kinematic platform by combining linear actuators with linear sensors for the external measurement of its position and orientation; the elongation of the linear actuators is obtained as a function of the movement of the platform. Linear sensors measure actuator elongations and provide these values as an input to the mathematical model.

### 2.2. Three-DOF Designs

**Figure 2.**
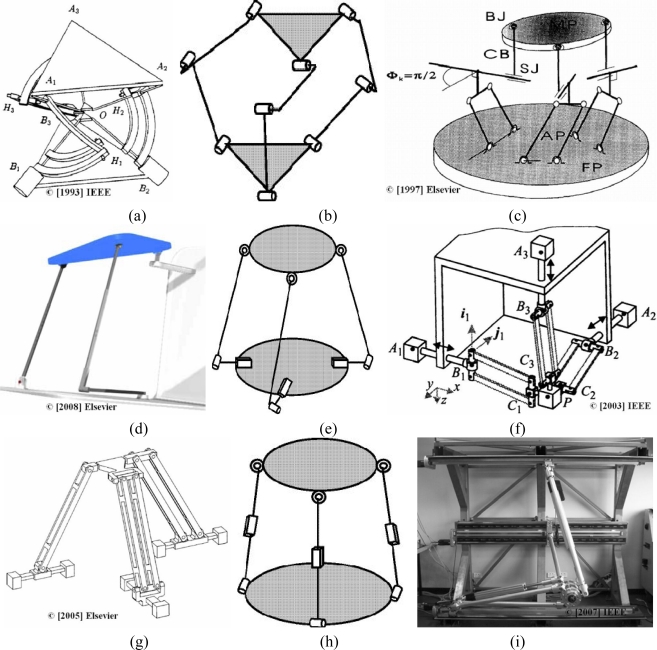
Three-DOF parallel robots: **(a)** Gosselin design (reprinted from [19] with permission from IEEE). **(b)** Tsai design [20]. **(c)** Ceccarelli design (reprinted from [21] with permission from Elsevier). **(d)** Gallardo design (reprinted from [22] with permission from Elsevier). **(e)** Carretero design [23]. **(f)** Ortoglide from Chablat (reprinted from [24] with permission from IEEE). **(g)** HALF-II from Liu (reprinted from [25] with permission from Elsevier). **(h)** Gallardo design [26]. **(i)** Tyapin design (reprinted from [27] with permission from IEEE).

There are multiple examples of 3-DOF parallel mechanisms, some of which are shown in Figure 2. Figure 2(a) shows a manipulator designed by Gosselin [19] where the active joints are revolution joints. The software developed to design the mechanism allows the interactive analysis of any spherical parallel 3-DOF actuated joint and the representation of the workspace, singularities and trajectories. Tsai [20] analyzed a translational platform with three identical kinematic chains [Figure 2(b)]. Each chain consists of an upper and a lower arm. Each upper arm is a planar four-bar parallelogram, and the two platforms are joined using revolution joints only. The axes of these revolute joints are perpendicular to the axes of the four-bar parallelogram for each chain. The mechanism constrains the manipulator output to translational motion and mimics the motion of the Delta robot moving platform. Cecarrelli [21] also designed a mechanism with three identical chains, but in this case, they contained a parallelogram. The connection of the chains was carried out by ball joints and prismatic guides to obtain suitable direct kinematics and easy actuation [Figure 2(c)]. Gallardo [22] analyzed a simple structure, with two legs and a spherical ball joint, which simplifies the study of the kinematic model [see Figure 2(d)]. This mechanism is not an overconstrained system which simplifies the study of the kinematic model. The results from the mathematical model were verified by comparing them to the results from a simulated parallel manipulator. Carretero [23] developed a manipulator with three identical PRS chains [Figure 2(e)].

The prismatic actuators lie on a common plane and have radial direction of action. The distal end of each actuator is joined to the lower end of a constant length leg by means of a passive revolute joint. The rotation axis of the revolute joint is perpendicular to the direction of the actuator and parallel to the horizontal base plane. The other end of each leg is attached to the moving platform by a passive spherical ball joint. The design variables minimize parasitic motion. This mechanism can be used in those applications that require elevation perpendicular to the base platform and pointing of a payload. Chablat [24] presented the Orthoglide [Figure 2(f)], a 3-DOF translational parallel system for machining applications. This mechanism, in which three fixed parallel linear joints are mounted orthogonally, provides a regular Cartesian workspace shape. The linear joints can be actuated by linear or rotary motors with ball screws. The end-effector is joined to the linear joints by three parallelograms. The chains are three identical PRPPaR legs. Other advantages of this design are uniform performances in all directions, low inertia, intrinsic stiffness and good dynamic performances. Liu [25] presented a manipulator with high rotational capability. This design utilizes a four-bar parallelogram which allows the output link to remain at a fixed orientation with respect to an input link. The moving platform is joined to the base by three non-identical chains. All joints that appear in the rotational DOF are with single DOF to obtain the high rotational capability [Figure 2(g)]. Moreover, the planar parallelogram, being as a leg, can improve the kinematic performance of the system, and this design allows us to obtain the desired number of DOF and to increase the system stiffness. In [26], Gallardo analyzed a family of 3-DOF manipulators with three identical RPS chains [Figure 2(h)]. The prismatic joints were actuated independently to provide the required number of DOF. Tyapin [27] proposed a new design optimization of the Gantry-Tau manipulator to avoid collisions between links and to maximize the reachable workspace [Figure 2(i)]. The mechanism consists of three-link arm, and two of them are mounted in a triangular constellation. Three linear actuators move the three arms independently. The developed algorithm allows us to obtain the internal link collisions. The author concluded that the design of the mechanism is crucial to avoid link collisions. The optimization step is based on the geometric descriptions of the workspace, unreachable area and the functional dependency of collisions.

### 2.3. Four-DOF Designs

**Figure 3.**
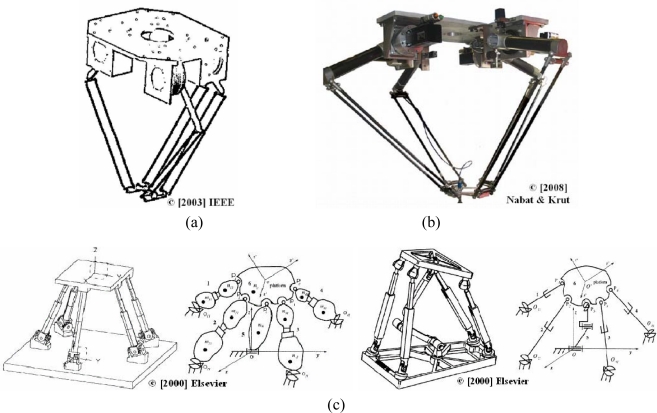
Four-DOF mechanisms: **(a)** Krut design (reprinted from [29] with permission from IEEE). **(b)** Nabat design [30] (reprinted with permission from Krut). **(c)** Wang analysis (reprinted from [31] with permission from Elsevier).

Four-DOF manipulators are extensively used for pick-and place applications. These mechanisms are divided into two important groups—mechanisms based on the Delta robot [12] and mechanisms based on the Scara robot [28,29]. Krut [29] developed a new parallel mechanism for Scara motions which produces three translations and one rotation about a given axis [see Figure 3(a)]. In [30], Nabat presented a parallel robot in which prismatic joints were replaced by revolute joints because of constraints caused by speed and acceleration, and it was considered that the dynamics can affect the design. The robot developed offers high speed while avoiding singularities [Figure 3(b)]. Wang [31] analyzed the static balancing of four types of spatial 4-DOF parallel mechanisms using counterweights and springs [Figure 3(c)]. The static balancing using counterweights consists in redistributing the link masses to maintain the center of mass of the mechanism fixed. Therefore, the weight of the links does not produce any torque or force at the actuators and the mechanism is statically balanced for any direction of the gravity vector. This is a great advantage for those portable mechanisms which can be mounted in different orientations. The static balancing using springs consists in selecting spring stiffness and locations. This method assures that the total potential energy of the mechanism is always constant. Therefore, the actuators do not have to support the weight of the moving links.

### 2.4. Five-DOF Designs

**Figure 4.**
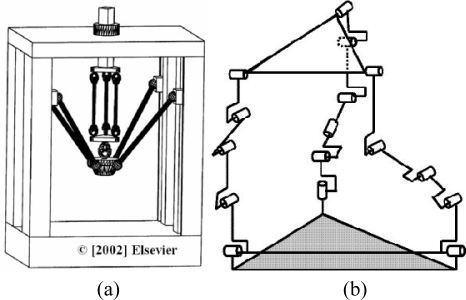
Five-DOF mechanisms: **(a)** Gao design (reprinted from [32] with permission from Elsevier). **(b)** Maurin design [33].

Few researchers have focused on 5-DOF spatial parallel mechanisms. Gao [32] proposed composite pairs and new sub-chains for the design of different mechanisms and presented a 5-DOF parallel manipulator utilizing composite limbs such as 4-PSS and a prismatic joint connected with a 3-UU chains. This 3-UU chain was connected to a spherical ball joint [Figure 4(a)]. In [33], Maurin presented a prototype for medical applications. This manipulator is based on revolute joints, and consists of three serial chains joining the base to the platform. The third chain is added to obtain the desired number of DOF, and only five joints are actuated [Figure 4(b)]. This mechanism offers mobility, compactness and accuracy around a functional point.

### 2.5. Six-DOF Designs

**Figure 5.**
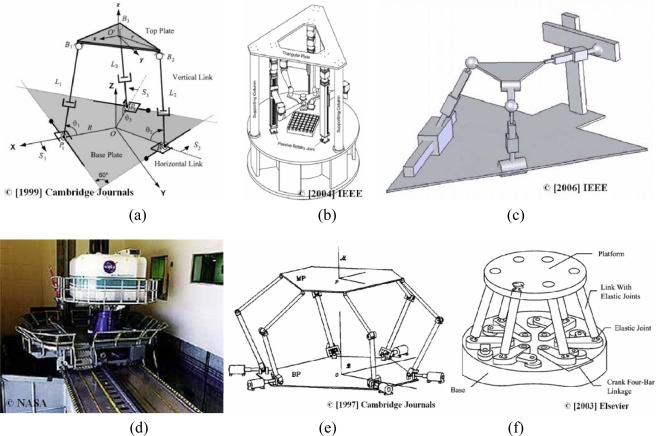
Six-DOF mechanisms: **(a)** Shim design (reproduced from [34] with permission from Cambridge Journals). **(b)** Yang design (reprinted from [1] with permission from IEEE). **(c)** Ben-Horin design (reprinted from [35] with permission from IEEE). **(d)** VMS (reprinted from [36] with permission from NASA. **(e)** Zanganeh design (reproduced from [37] with permission from Cambridge Journals). **(f)** Wang design (reprinted from [38] with permission from Elsevier).

There are numerous examples of 6-DOF mechanisms in the literature. Many of these designs are based on the Stewart platform and try to improve its performance. The design proposed by Shim [34] is intended to be used as a wrist of a robot [see Figure 5(a)]. This mechanism consists of linear actuators and optical position sensors. The position of the actuator is measured by an optical sensor. This sensor consists of a laser diode, two mirrors and a position sensing device (PSD). The position sensing resolution of the device is approximately ±5 μm. A force sensor is installed at each actuating link, reading up to 10 N with a resolution of 2.22 × 10^−4^ N, to reflect accurately the applied forces on the mechanism links. The manipulator is controlled by using the position sensor readings of the links. These readings are the inputs of the model. The system offers high precision and the capability of pure rotation generation, and it is easy to predict the moving platform motion. Yang [1] designed a parallel mechanism with three identical RPRS chains. This mechanism has the joint axes, except the three spherical ball joints at the chain ends, parallel to each other and perpendicular to the base plane in order to decouple motion. This system offers high stiffness in the vertical direction [Figure 5(b)]. Ben-Horin [35] obtained the 144 different structures of a 6-DOF mechanism having three identical kinematic chains. Considering different combinations of chains, this number is larger than 500,000 [Figure 5(c)]. Figure 2(d) shows a rotary 6-DOF mechanism designed by NASA to simulate vertical movements (VMS) [36]. In [37], Zanganeh studied a manipulator with six kinematic chains, connected to the base by means of universal joints and to the moving platform via spherical ball joints. Each chain consists of two links connected by a revolute joint and actuated by a motor at the base to reduce the leg masses and inertias [Figure 5(e)]. Wang [38] developed a parallel manipulator with elastic joints to obtain high precision. The system consists of six chains, and each chain is made from four-bar linkages [Figure 5(f)]. The elastic joints provide the mechanism with highly precise operation.

**Figure 6.**
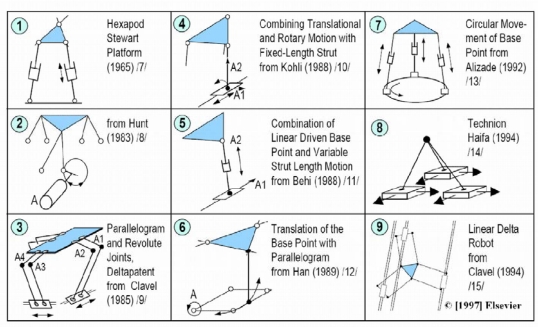
Known hexapod systems (reprinted from [39] with permission from Elsevier).

Pritschow [39] summarized, in Figure 6, some of the most famous hexapod designs, and developed a methodology for the design of parallel mechanisms.

### 2.6. Sensor Application of Parallel Mechanisms

Parallel mechanisms are also used in the kinematic structure of several types of sensors. A Stewart platform can be used as a wrist force sensor, where six-axis units (multi-axis force-torque sensor) measure the three Cartesian coordinates: X, Y and Z. Gaillet and Reboulet [40] developed an isostatic six component force-torque sensor based on the octahedral structure of the Stewart platform. A review of the first force-torque sensor designs based on parallel mechanisms can be found in [41].

In recent years a number of specialized papers have presented new designs based on the Stewart platform. Dwarakanath [42] designed a force-torque sensor to achieve well-conditioned transformation between the input and output forces. The legs acting as elastic elements were designed as a transducer to have resisting and restoring characteristics when dynamic load is applied. The design provides sensitivity, small sizing and manufacturing simplicity.

Yao [43] analyzed a pre-stressed six-component force-torque sensor and determined the key structural parameters of the sensor to obtain high measurement sensitivity, good isotropy and least effect of frictional moment. The author concluded that the errors in the measurement, obtained from calibration data, can be due to machining or assembling errors and joint frictional moment.

Sui [44] developed a static measuring model of a force-torque sensor in which the gravity of the links is considered. The sensor consists of a top and a base platform and six links. A link contains a single-axis bidirectional force transducer and two link rods, and finally, two spherical ball joints connect the links with the platforms. The developed model is a generalizable model where the link and top platform weight is compensated. To verify the model, the force is measured by applying loads to the sensor in different directions.

Chen [45] presented a six-axis force-torque sensor and derived the analytical equations to obtain high sensitivity isotropy and sensitivity. These characteristics are determined by four parameters: the radius of the upper platform, the radius of the lower platform, the length of the link and the position angle difference of the two platforms. The experiments show that when the radius of the upper platform, the radius of the lower platform and the length of the link are simultaneously increased or reduced, the torques will be changed, but the sensitivity isotropy, sensitivity of force and the shape performance will remain constant.

Frigola [46] designed and analyzed a touch pad based on a parallel platform. The developed model obtains force and torque feedback by means of sensor readings of the leg forces. This work demonstrated how the integrated effect of the dry friction, in the twelve spherical ball joints, degrades the static measurements and how the mechanical resonance degrades the dynamic ones. To use a structure with a self-stress could solve these problems.

## 3. Kinematic Model

The *mathematical model* of a mechanism can be divided into two phases: the kinematic model and the dynamic model. Some authors have called these phases the geometric and the dynamic model [47,48], although in this paper we will use the first nomenclature because it is widely used in the specialized literature.

The *position kinematic model* establishes mathematical relations between actuated joint coordinates of the mechanism and the end-effector pose. The end-effector position is defined by its spatial position and orientation with respect to a global reference system. The local relations between two successive reference systems are expressed in function of variables which allow us to describe every change in the end-effector position and orientation.

The *differential kinematic model*, or velocity kinematic model, obtains the relations between the velocities of the joint movements and the velocity of the end-effector, and it is expressed by means of the Jacobian matrix.

The *dynamic model* obtains the relations between the generalized accelerations, velocities, coordinates of the end-effector and the joint forces. This model analyzes the influence of forces, inertias, gravity, torques and non-geometric effects due to friction, gear transmission or backlash. Some authors [49–57] have considered the influences due to the mechanism dynamics in the kinematic model, absorbing dynamic errors of the mechanisms by means of the non-geometric parameter calibration.

The first known studies on parallel robot kinematics were performed by Fichter [58] and Merlet [59]. Fichter analyzed the Stewart platform, determined the equations to obtain the leg lengths, directions and moments of the legs and derived these equations. To obtain the force and the torque of the manipulator he developed the dynamic analysis, but he assumed that the legs are massless and exert pure forces. Merlet developed the Jacobian, derived the dynamic equations and determined the workspace.

The initial position problem has two different phases:

1. Obtaining the non-linear equations that relate the joint variables and the end-effector position and orientation, thereby arriving at the forward and inverse position kinematic model
2. Solving the non-linear equation system obtained in the previous step

Depending on the approach to the problem, there are three important groups:

- Graphical methods
- Analytical methods
- Numerical methods

Graphical methods are especially used in simple mechanisms, and they can be divided into three subgroups. Dyadic decomposition methods [60] allow us to obtain the mechanism position by means of a compass and a ruler. Interpolation procedures [60] belong to the second group of graphical methods. And, finally, the modular approach methods [61], which decompose the mechanism into modules that can be independently analyzed.

An analytic approach is used in the analytical methods, although the solution procedure is usually numerical. There are three methods to obtain all the solutions: polynomial continuation methods, elimination methods and polynomial Gröbner bases.

The polynomial continuation method [14,62,63] is a numerical method that finds all isolated roots of a polynomial system from a known initial solution. The elimination method [14,26,62,64] is analytical, but usually the polynomial must be solved by a numerical method. In Gröbner bases [14,65,66], an invariant polynomial is obtained. Each subsequent equation adds at most one variable. The invariant polynomial is solved to find all possible values of one known. The other equations obtain the values of the other variables for each solution. The methods described present a high computational cost, so the Newton-Raphson method is used to obtain only one solution.

Computation methods are based on systematic algorithms that allow us to automate the analysis of the kinematics of the mechanisms, independently of the number of DOF or complexity of the mechanism. For instance, programs based on multibody systems [67,68] are used to model the behaviour of interconnected rigid or flexible bodies. These bodies may suffer large translational and rotational displacements. Although this method is mainly used to solve the dynamics of a mechanism, it can also be used to solve the kinematics [69]. A multibody system models a mechanism by means of coordinates that define the position of all their elements in a univocal way. Constraint equations from kinematic pairs are applied on the mechanism elements. To solve the problem, one of the initial pose problem solutions must be obtained first, and then the model has to be checked for redundant constraints. Secondly, the equations that derive from the redundant constraints are eliminated. Finally, finite elements analysis is performed, using for example the Newton-Raphson algorithm to solve the non-linear system.

The position kinematic model can be solved by the direct or inverse kinematics, depending on the input and output variables.

The *direct position kinematic model* (DPKM) is used to calculate the position and orientation of the platform, given the values for the joint variables of the mechanism, according to Equation 1:

(1) $${\left[x,y,z,\alpha ,\beta ,\gamma \right]}^{\prime }=g\left(q_{1},..,q_{n}\right)$$

The *inverse position kinematic model* (IPKM) is used to calculate the mechanism’s joint variables, given by *(q*_1_, .., *q_n_)*, for a position and orientation of the platform, *(x*, *y*, *z*, *α*, *β*, *γ)*, according to Equation 2:

(2) $$q_{k}=f_{k}\left(x,y,z,\alpha ,\beta ,\gamma \right)\;\text{with}\;k=1..n$$

The differential kinematic model is usually used to determine singular configurations or to control the mechanism.

The *direct differential kinematic model* (DDKM) is used to obtain the velocity of the end-effector, *given* the joint velocities.

The *inverse position kinematic model* (IDKM) is used to obtain the joint velocities, given the velocity of the end-effector

The inverse kinematics of closed-chain mechanisms can be solved through geometric [13], analytical [13,70–73] or applying the Denavit-Hartenberg (D-H) model [74,75], where the solution is usually obtained by numerical methods such as the Newton-Raphson algorithm. Geometric methods can be used in simple systems. The Newton-Raphson method is very sensitive to the initial position introduced in the algorithm. Therefore, if this position does not draw near to the solution of the system, the algorithm cannot converge. Rao [76] proposed the use of a hybrid optimization method starting with a combination of genetic algorithms and the simplex algorithm. This method carries out a global search of the initial solution with genetic algorithms and, subsequently, uses the simplex method for the local search.

To solve the direct kinematic problem, the use of analytical methods is complex, given that the chains share the same unknown factors; therefore, the most suitable resolution methods tend to be numerical [41,71]. However, for systems with 2-DOF a geometric or analytical solution may be easier to obtain and more efficient.

Many studies have developed methods to obtain a mathematical model which allow us to solve the direct kinematic of parallel mechanisms. In [77], Innocenti solved the direct position analysis and found all the possible closure configurations of a 5-DOF parallel mechanism and in [78], the author analyzed a 6-DOF fully parallel mechanism. The developed method finds out all the real solutions of the direct position problem of a 6-DOF fully parallel mechanism. This method determines the roots of one equation, representative of the direct position analysis, in only one unknown.

Merlet and Bonev [79,80] suggested using sensors to solve the direct model. Merlet demonstrated that the measurement of the link lengths is not usually sufficient to determine the unique posture of the platform, and that this posture can be obtained by adding sensors to the mechanism. Sensors can be added by locating rotary sensors in the existing passive joints or by adding passive links whose lengths are measured with linear sensors. Although the first solution offers less interference between the links, the mechanism motion may be reduced. Two sensors on each of these joints allow us to measure the direction of the link. The position of the other extremity of the link may be calculated with the link length. A unique solution is obtained by adding four sensors on the passive joints, for a general case. Three sensors are sufficient for a particular mechanical architecture.

Bonev solved the direct kinematic problem of parallel manipulators by adding three linear extra sensors. Linear Variable Differential Transformers (LVDTs) and Cable Extension Transducers (CETs) are linear extra sensors commonly used in parallel mechanisms. The LVDT sensor has a low measurement range (0.5 m), it requires support electronics, its installation requires universal joints and its price is high. The cable end of a CET is joined to the moving object and the CET is fixed. The cable extends or retracts when the object moves. The CET produces electrical signals proportional to the movement of their extension cables. And the linear displacement is converted to angular displacement with the cable being wound onto a cylindrical spool. A rotary sensor measures the spool rotation. The range provided by these sensors is from 0.04 to 40 m, the accuracy is about 0.02% of full scale for potentiometers and 0.02% for shaft encoders and the repeatability is 0.02% of full scale. The sensors selected to this design were the CETs, and they connect the planar base and the planar moving platform at distinct points. The linear extra sensors were implemented considering sensor misalignment range, link sensor interference and singularity of a matrix which depends on the geometry of the moving platform and the arrangement of the sensor base attachment points and base joints. To solve the model, three coordinates are obtained directly from the extra sensory data. In [75,81], the authors solved the kinematics of the system by considering each of the mechanism’s legs as an open chain. Some authors [82,83] have studied special configurations of the Stewart platform obtaining that the explicit expressions for these configurations can offer the geometric limitations to motion. Ball [84] announced another methodology based on screw theory and developed by Hunt [11]. The velocity fields of systems of interconnected bodies are described by systems of instantaneous screw axes, and the static force systems acting on the rigid bodies are described by systems of wrenches, which are vectorially homogenous to the screw systems. Utilizing Ball’s reciprocity relationship, it is possible to relate the two types of vectors, and an instantaneous screw axis can be represented by a vector pair [85]. This theory allows us to obtain and analyze Jacobian matrices and is based on Kennedy–Aronhold centre theory and on Chasles theorem. Kennedy–Aronhold centre theory demonstrates that if the instant rotation center of a body is known, velocity can be obtained by multiplying its angular velocity by the distance between the instant rotation center and the body. Chasles theorem explains that any motion of a rigid body can be achieved as a rotation around a geometrical line together with a pure translation along this line, known as the screw axis of the motion. In [86], the direct kinematics for a 3-DOF manipulator is solved by means of screw theory, obtaining four possible solutions for this mechanism. This methodology is very useful when solving velocities and accelerations, but classical methods are usually more convenient for obtaining only the position problem. In [26], the position problem is solved by means of classical methods and screw theory is used to obtain the mechanism dynamics.

Subsequently new contributions appear, trying to search for all the direct kinematic solutions, based on a new concept, the multibody system [87]. A multibody system is used to model the dynamic behaviour of interconnected rigid or flexible bodies that can move relatively to each other. This method can be applied in two different ways [68]. The first way utilizes reference point (or Cartesian) coordinates. The derivatives of the position coordinates of the centre of mass of the link and the orientation parameters of the link are used. The velocity equation is determined through the constraints imposed by the joints. The second way uses fully Cartesian (or natural) coordinates. In this case, the position of the nodes on joints and unit vectors on joint axes are used. The kinematic parameters are nodal velocities and derivatives of unit vectors, and they are related through derivatives of length restrictions and joint constraints. In [88], the author extended the kinematic manipulability to general constrained rigid multibody systems.

In recent years computational intelligence, such as artificial network, genetic algorithms or fuzzy logic, is becoming important in solving mechanisms.

Artificial intelligence is concerned with intelligent behaviour in machines, and it involves perception, learning, reasoning, communicating and acting in different environments.

An artificial neural network is a mathematical model that tries to simulate brain hardware structure and reproduce its low level capabilities, such as pattern recognition or data classification, through a learning process. It consists of an interconnected group of artificial neurons that process information. This method presents the capability to derive meaning from imprecise data or complicated systems, so it is used in complex mechanisms where there is not enough data or it is very difficult to obtain the kinematic equations by means of classical methods. Other advantages of these artificial systems are [89,90]:

- *Adaptive learning*: The capability to learn to do tasks based on the data given for training or initial experience.
- *Self-organization*: The ability to create its own organization or representation of the information.
- *Fault tolerance*: The structure is degraded when a partial destruction of a network takes place. However, some network capabilities may be retained.
- *Real time operation*: Computations in a neural network can be carried out in parallel in a special device.

Fuzzy systems try to reproduce high level capabilities of the brain, such as approximate reasoning, because the capabilities are usually non precise or fuzzy in the real world [89]. This method allows us to make decisions with imprecise and incomplete information. Some of these advantages are that computations are very simple and allow us the use of non precise terms in the rules. On the contrary, it is difficult to estimate the membership function and there are many ways of interpreting fuzzy rules. Thus, this method is used in unknown and complex environments.

Different systems that apply these methods can be found in literature. In [91], a multiple neural network structure, called CMAC (Cerebella Model Arithmetic Computer), was developed to solve direct kinematic problem of the Stewart platform. This method offers a considerable time saving in comparison to the neural network models developed until then and allows us to easily obtain a model which provides an approximation to the solution problem. However, it can only be applied directly to those configurations for which it has been designed, since the model would have to be modified for each new design. Output variables can be obtained by means of input variables without knowing the other system variables as occurs in models based on neural networks. Although a priori represents a simplification, the system calibration usually requires the understanding of the mechanism behaviour.

**Figure 7.**
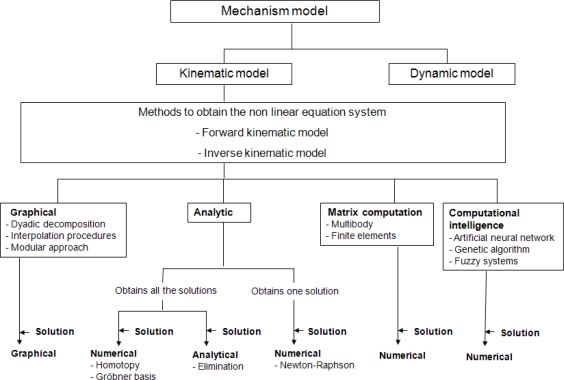
Scheme of different methods to modeling and solving parallel kinematic mechanisms.

In [92], Sadjadian has compared the accuracy of three numerical methods (neural network, quasi-closed solution and 4th Taylor expansion) to solve the forward kinematic problem of a redundant parallel manipulator. The author has concluded that the 4th order Taylor series approximation offers the best prediction errors of the three. Figure 7 shows a scheme with the different methods analyzed for modelling and solving equation systems of parallel kinematic mechanisms.

### Classical methods or computational intelligence?

In this section it has been shown that neural networks and fuzzy systems have important advantages, and they have been applied in different areas such as robotics. These methods can be suitable for those systems where it is very complex or impossible to obtain the model by means of classical methods, for example, in very complex mechanisms, non well-defined problems, when the environment is unknown [89] or in those mechanisms where there is a behaviour model for trajectory programming tasks [90]. However, in those devices in which a complete performance knowledge requires a careful kinematic analysis, computational intelligence allows us to determine inputs and outputs, but it is not possible to know the value of the rest of the parameters due to their non-parametric nature. This fact makes it difficult to relate geometric and non-geometric parameters. In these situations, it is more suitable to solve the kinematic problem by means of the classical methods.

## 4. Calibration

### 4.1. Principles

Robot calibration consists of identifying the geometric parameters in order to improve the model accuracy. In parallel mechanisms, the objective is to reduce the end-effector position error by means of an accuracy identification of the kinematic parameters. This procedure allows us to obtain correction models to establish corrections in the measurement results. Moreover, the calibration procedure quantifies the effects of the influence variables in the final measurement. The steps to achieve this goal can be divided in five phases: determination of the kinematic model by means of non-linear equations, data acquisition, optimization or geometric parameter identification, model evaluation and, finally, identification of the error sources and implementation of correction models.

The first step, determination of the kinematic model, consists of obtaining the non-linear equations that relate the joint variables with the position and orientation of the end-effector and the initial values of nominal geometric parameters.

The second step is data acquisition. The home position is a position, within the robot working range, where all joint angles have a pre-defined value. The displacements of the end-effector are usually measured with respect to this defined position.

The following step, optimization or geometric parameter identification, is usually carried out by means of approximation procedures based on least-square fitting.

Once the optimization is applied, an evaluation of the model in different positions than those used in the identification process must be carried out to test the model obtained.

Finally, an identification of the error sources and a modelling and implementation of the correction models can be performed.

In [93], Everett pointed out the differences that exist between the calibration methods for open-loop mechanisms and closed-loop mechanisms. Although in both cases, the objective of the calibration is to minimize the error between the measured pose of the end-effector and the calculated pose, in parallel mechanisms special care must be taken, since it is not possible to choose the model parameters arbitrarily. This is mainly due to the fact that some parameters are inter-related as they belong to a closed chain. This characteristic in parallel robots requires two types of equations in the calibration method. On one hand, those transformations that relate the end-effector location with the reference system of the base by means of open-loop kinematic chains, and on the other hand, those closed-loop transformations which contain the constraints imposed by the closed-loop chains.

### 4.2. Calibration Procedure

As is known, the calibration procedures present three well differentiated levels, level one calibration or joint level calibration, level two or kinematic calibration and level three or dynamic calibration [94,95].

#### 4.2.1. Joint Calibration

The joint calibration consists of determining the relations between the signal produced by the joint displacement transducer and the actual joint displacement. By means of this modelling, two more parameters are added for each joint in the mathematical model of the mechanism. For prismatic joints, d_0i_ (joint displacement in the model initial pose, with respect to the sensor reference mark) and k_i_, (function curve of the sensor output). In case of rotary joints, these parameters are θ_0i_ (joint rotation in the model initial pose, with respect to the sensor reference mark) and k_i_. At this level, data acquisition is performed by means of some external measurement devices to determine the actual joint angle accurately, or by moving the joint to any known configuration. Usually, easily measureable configurations are chosen. In open-loop mechanisms, configurations in which several elements are aligned are frequently used. In closed-loop mechanisms, due to their geometry, those configurations with a known joint angle are more suitable. In this case, the forward model is required, and in closed-loop mechanisms this problem presents more complexity. Another possibility is to place the end-effector in a known position and orientation belonging to the workspace, in order to solve the problem by means of the inverse kinematics. The equations developed in the modelling phase allow us to carry out the parameter identification process. The correction phase ensures that the parameter values are precise, using a controller to convert the signal that comes from the joint transducer into a representative value of the actual joint angle. In [96], Sommer described how to model the behaviour of the joint sensor, according to prismatic or rotary joints, by introducing two more parameters for each joint in the mathematical model of the mechanism. The aim of this first calibration level is to split up the error sources and to maintain the relation between the physical and mathematical parameters during the calibration process.

#### 4.2.2. Kinematic Calibration

The kinematic calibration consists of determining the kinematic geometry of the mechanism and the correct joint angle relationship.

(a) Kinematic model

Just like in the joint calibration, in this level the first phase is to determine the kinematic model. This model allows us to obtain equations which relate the joint variables of the mechanism with the position and orientation of the end-effector.

It is not an easy task to obtain a suitable model that ensures the optimal accuracy of the system, and one of the unsolved problems in this working area is that it is very difficult to obtain a generalizable model – it is specific to the design under study.

In a parallel mechanism, the end-effector position is limited by certain restrictions. Calibration will obtain this position for several poses of the end-effector or configurations. Constraint equations are a function of m geometric parameters of the robot, of the measurements obtained at the position and of the n pose parameters of the process of calibration. For N calibration poses, there are (N × c) constraint equations with (m + N × n) unknowns, and the number of constraint equations must be greater or equal to the number of unknowns. The solution to this non-linear system is usually obtained by means of numerical methods, such as minimizing the sum of squares of the constraint equations.

Everett divided kinematic calibration models into two categories [97]. Models belonging to the first category assume that all the joints in the mechanism can be modeled as revolute or prismatic joints. To be able to make this assumption, the kinematic model should fulfill three characteristics: complete, equivalent and proportional. In these models an objective function is usually minimized, and the characteristic of proportionality is very important to guarantee numerical stability.

The second category includes those models in which it is considered that some of the joints can contain higher pairs. In these models, besides revolute and prismatic joints, some additional movements can appear and must be expressed as a function of the variable joints. These models add an offset to each joint, adding three new parameters to each one. This fact originates a multitude of possible functions to model the joint, and the concepts of equivalent and complete model are not applied to this category.

##### What type of errors can appear and how do they influence?

To attain a high level of accuracy, the model must consider the most significant geometric and non-geometric parameters for the mechanism designed.

Geometric errors

Geometric errors may appear from manufacturing errors or from the deviation of the offsets of the components. Joints in the links are not perfect, so the axes cannot be perpendicular between them, and they cannot intersect in the exact center of the joint. Errors when assembling actuators can cause the axis of each actuator not to pass through the center of the joint. Other errors can appear when measuring the offset of the components at the location of the mechanism’s joint.

One of the most widely used geometric methods for modelling an open-loop or a closed-loop mechanism is the well-known Denavit-Hartenberg method [98]. This method allows us to model the joints with four parameters. One of the limitations of this method appears when it is applied to those mechanisms that present two consecutive parallel joint axes. In this case, an infinite number of common normals of the same length exist, and the location of the axis coordinate system may be made arbitrarily. In [97], Everett mentioned the most relevant publications which propose some solutions for this limitation. In [99–102], the authors developed methods to obtain a complete, equivalence and proportional model. Some research has been carried out on obtaining an accuracy model [103,104], in which manufacturing tolerances, assembly errors and offsets are studied to develop an algorithm for the identification of the kinematic parameters of the Stewart platform. In each joint–link chain three types of parameters appear: measurable variables for describing the extension of the prismatic joints, un-measurable variables describing joint angles and geometric parameters describing the dimensions of the platform.

Once the kinematic model has been determined the number of parameters will be fixed, and will depend on the selected method. Wang [103] determined that the number of geometric parameters to define a kinematic chain is 22 [103], and this number can be reduced to 7 if passive joints are considered as perfect joints. Kinematic parameters usually correspond to the position of spherical, universal or revolute joints. Therefore, each spherical ball joint has three kinematic parameters, and each prismatic joint adds a new parameter, corresponding to its elongation [105]. The method employed to calibrate the Stewart platform establishes the orientation constraint by maintaining two attitude angles of the end-effector constant.

Non-geometric errors

The non-geometric errors can appear from backlash, gear transmission, friction, gravity, temperature or compliance [106]. The non-geometric models try to predict and compensate these errors.

Some authors [107] have developed non-geometric models to achieve this. In [108], Renders has gathered the influence of non-geometric errors. The errors that have the most significant effect on accuracy are joint flexibility, link flexibility, gear transmission error, backlash in gear transmission, and temperature effect. Flexibility in joints and in links causes 8% - 10% of the position and orientation errors of the end-effector, and link flexibility is usually 5%. Joint flexibility errors can be reduced by mounting the joint encoders directly on the joint after the transmission units, instead of mounting them on the motor shaft. Gear transmission errors are mainly due to runout and orientation errors. The contribution of backlash is from 0.5%–1%. The error due to temperature effects causes 0.1% of the total error. Calibration is usually performed in an environment where the temperature is controlled. Next, a correction model that considers the working temperature is applied. Judd [109] developed a model to correct problems with robot accuracy resulting from imperfections in the main spur and encoder pinion gears, errors in the link and joint parameters and structural deformations. Hollerbach [110] introduced a calibration index that considers sensed and un-sensed joints and single and multiple loops. In [111], Gong developed an algorithm for non-geometric error identification and compensation by means of the inverse calibration of the system, analyzing the effect of geometric errors with temperature variation and compliance. These methods separated the influence of the errors due to geometric and non-geometric parameters, in order to optimize geometric parameters by means of a traditional static calibration, and to model and correct non-geometric errors by means of a dynamic calibration. However, they are not generalizable and they do not allow us to know the individual influence of each non-geometric component.

##### How many parameters are necessary in the kinematic model?

To consider all the possible errors in the same kinematic model is a laborious task that, due to the complexity of the model, does not always increase the accuracy of the result. Moreover, it can add errors in the resolution of the problem, for example in the case where the model adds discontinuous functions for backlash or gearing errors, or when parameters are a function of joint variables instead of being a function of constants.

It is not possible to know a priori what parameters must be used in the calibration process to obtain the desired accuracy, but the mechanism repeatability must be considered in order to predict the order of magnitude of the accuracy that can be reached.

Everett [112] analyzed models based on forward kinematics and explained that there are a maximum number of parameters that must be identified, and that the model accuracy cannot be improved by adding extra parameters. The author explained that unless all joints are moved, not all parameters can be identified, because if one or more joints do not move, some unknowns can be decomposed, shifted and absorbed into others. He determined that four parameters must be considered for each revolute joint, two of which must be orientational. And for a prismatic joint two orientational parameters are necessary, applied about the non-colinear axes before and perpendicular to the translational joint axes. Thus, it is more frequent to add parameters to compensate non-geometric errors in the geometric model [113].

Regardless of the method selected, calibration can be solved by means of inverse or forward kinematics. The calibration problem can be formulated in terms of residual measurements. For example differences between the joint variable measurements and the values obtained by the inverse kinematic model. This model offers significant advantages compared to the one based on forward kinematics, since calculations in the latter case are more complex and require more time to be solved. Besides, the solution in the inverse model is unique, unlike the forward one in which several possible solutions can appear. The inverse model allows us to decouple the calibration problem for every kinematic chain, and the constraints can be expressed by analytical equations. This method gives numerical efficiency but that measurement of the positions has to be very precise.

Another possibility is to perform partial measurements of the position, posing the problem in terms of errors between measured values and computed values via forward kinematics. In [114], the kinematic model was solved using this method.

In [103], Wang presented a method for the calibration of a 6-UPS robot that uses parallel kinematics according to the lengths of kinematic chains and positioning parameters of the platform. Zhuang [115] developed a model in terms of residual measurements of the difference between the measurement length of the kinematic chain and the one obtained from the model without using forward kinematics. The author solved the problem by means of minimizing the sum of squares of the constraint equations. Parameter errors are mainly due to errors in the assembly of spherical and universal joints, and solutions obtained for some parameters are out of the range provided by the method, which means that some constraint equations are not satisfied. In [116], Daney developed an algorithm to perform the calibration by means of partial measurements of the position, thanks to the elimination of the rotation parameters. This algorithm endeavours to obtain the advantages of the two models, inverse and forward, combining the elimination of symbolic variables through numerical optimization, which allows us to obtain numerical stability. The method is applied for three different cases in which the number of restrictions and equations to perform the calibration varies. The conclusions are that methods that obtain better results do not match those which have a higher algebraic computational cost, and that the elimination of the rotation parameters improves the accuracy of initial estimations. In [117], the kinematic problem was solved by equalizing constraint equations to a value, *ɛ_i_*, to obtain a solution.

###### Data acquisition

This is probably one of the steps with more unresolved questions, mainly due to the difficulty in finding a general methodology. Therefore researchers try to find the best procedure, usually applied to a specific mechanism. This subsection shows how different authors have dealt with these unsolved problems.

Any measurement error of the external instrument is propagated to the results of the identified parameters. Therefore, it is recommended to use an instrument for data acquisition that is, at least, one magnitude order more accurate than the mechanism whose parameters are going to be identified.

The ability to measure the global reference system of the mechanism which is going to be calibrated usually determines the different options of data acquisition and the sensors that are going to be used at this step.

If the global reference system can be measured by means of an external measurement instrument (for example a laser tracker or a coordinate measuring machine), a direct geometric transformation can be established. This transformation obtains the coordinates of the measured points in the global reference system of the mechanism. In this case, direct comparisons in the objective function, between measured data (or their geometric composition) and mechanism model nominal data, can be made providing both are expressed in the same reference system.

Unfortunately this relation is not usually easy to obtain through a direct measure. In these cases, least-square methods can be used with a finite set of data. These methods allow us to obtain an approximation of this transformation, which depends on the mechanism error in the points and configurations used in data acquisition. Moreover, this approximation is absorbed by the objective function. For that reason, it has direct influence on the value of the identified parameters. This method is therefore not suitable for parameter identification procedures in which positioning accuracy is mandatory or when it is necessary to generalize the positioning accuracy obtained in the identification process to other areas of the workspace.

For all these reasons, the geometric relation between the reference system of the measurement instrument and the mechanism global reference system must be established accurately. Otherwise, the objective function should be obtained starting from a reference position and evaluating Euclidean distances between datasets.

##### How can we measure?

Classical robot calibration methods use additional sensors to measure the position and orientation of the end-effector and the joint variables of the ball joints, where the calibration process optimizes the error between the measured and computed variables.

The type of sensors used in a parallel mechanism affects not only the design process but also the calibration procedure. Sensors can be used to measure the variables of the mechanism, usually the active ones, in order to obtain the necessary data to solve the kinematic problem. In the calibration procedure, mechanism internal sensors are used to obtain information of the system. These data will be the input to the mathematical model. The output of the forward model will be the calculated position and orientation of the end-effector. In the calibration procedure, the nominal and the calculated position and orientation of the end-effector are compared and the mechanism geometric parameters are obtained. On the other side, external devices having measurement systems allow us to measure the nominal position and orientation of the end-effector. It is important to note that every measurement error of the measurement device will be propagated to the calibration results. Therefore, measurement devices must be more accurate than the desired accuracy of the mechanism that is going to be calibrated.

In parallel mechanisms, the most used internal sensors are lineal optical sensors (for measuring the elongation of the actuator), rotary optical sensors (for measuring the motor rotation of the actuator), linear variable differential transformer (LVDT) and force-torque sensors (for the dynamic calibration).

Accuracy of linear and rotary optical sensors is highly dependent on the method used to couple the encoder to a shaft. This value can commonly reach ±0.5 μm and ±1 arcsecond, respectively, and resolution 1 nm and 0.02 arcseconds, respectively. LVDTs present a very high reliability. Accuracy and resolution are limited only by the signal conditioning electronics and the analog-to-digital converters. Resolution can reach the nanometer range. These types of sensors are used to measure relative motion between objects whose surfaces only move a little bit with respect to each other. Besides, their measurement range is low (about 0.5 m). On the contrary, linear optical sensor measurement range is up to 30 m and rotary sensors offer not rotation limit for incremental encoders and several turns for absolute encoders. Force-torque sensors are commonly used to measure the applied forces on the mechanism links. These devices frequently present a force sensor accuracy of 6 mN and a torque sensor accuracy of 30 mN·mm

External devices typically used in the calibration procedure to improve the mechanism accuracy are cameras, laser trackers, coordinate measuring machines (CMM) or autocollimators. Cameras and autocollimators are non-contact measurement instruments. These devices are therefore more suitable when the influence of measurement forces can affect the results. Cameras and 3D imaging sensors present compactness, robustness and flexibility [118]. The rapid development of these devices in the last decades has significantly improved their accuracy. Another advantage of these sensors is their portability. Moreover, the recent development in this technology allows us to perform a massive data acquisition. CMMs offer high resolution and accuracy (about a few micrometers). The laser tracker volumetric accuracy is about tens of micrometers. A typical measurement range is about 900 mm × 1,200 mm × 700 mm in a CMM and up to 40 m radius range in a laser tracker. Therefore, these last devices are suitable for large parallel mechanisms. Although autocollimator resolution (0.1 arcsecond) and accuracy (0.2 arcseconds) is very high too, the measurement range is very low (from arcseconds to a few degrees). By contrast, measurements with a CMM take a lot of time. In order to obtain a high accuracy and efficiency, optical and contact sensors are used in combination. A visual sensor provides global information of the object surface. On the contrary, force and tactile sensors obtain local information. In recent years, several studies have focused on the combination of visual and force/tactile sensors to obtain a high knowledge of the mechanism behavior [119].

Everett [120] designed a sensor for measurements in the calibration of mechanisms. This sensor did not apply external constraint forces on the mechanism. The sensor used three LED/phototransistor pairs as optical switches. Each switch used a LED that shone through an optical fiber. The light left the fiber and passed through a gap. On the other side, another fiber received the beam, and a phototransistor received the light. This work described the sensor construction, the sensor performance and calibration. The sensor was installed in the gripper of a robot as a tool. Precision spheres, having a diameter of 12.7 mm, were mounted in the workspace of the robot. The spheres were located within an area of 1,600 cm^2^. Firstly, the relative positions of the spheres were measured with a coordinate measuring machine. Secondly, the sensor was positioned over these spheres automatically. Three light beams defined position with respect to the sensor. Two beams were separated 10 mm and the third beam was mounted 30° away from them. The robot was programmed to find the trip point of the sensor. This point defined the origin of the sensor coordinate frame. Then, the sensor input was examined to determine which light beams were broken. The test obtained 100 trip points and the measurement error was calculated as the standard deviation of the position errors. The measurement error was 0.06 mm and the repeatability was between 0.06 and 0.08 mm. To calibrate the mechanism by means of the developed sensor, the phases are the following:

- To develop a model that relates measurable joint positions to mechanism pose
- To measure a sufficient set of joints positions and their corresponding mechanism poses
- To identify the parameters of the model
- To determine the spheres centre relative to the fixture datum (for example by the coordinate measuring machine)
- To collect calibration data by the sensor.

A widespread classification in the kinematic calibration of parallel robots is the one presented by Merlet in [13]. Calibration models are classified into three types: external calibration, constrained calibration and auto-calibration or self-calibration.

Although the simplest way of obtaining the necessary data is by using internal sensors, their assembly is difficult in most of the systems. In external measurement systems, it is usually necessary to establish, in an approximate way, the relation between the measurement system and the reference system of the end-effector. And this procedure has the problems described above.

##### Self-calibration

In self-calibration methods, additional sensors are added to passive joints and each pose of the mechanism can be used as a calibration pose. These methods require that the number of internal sensors is greater than the number of DOF of the mechanism. Self-calibration methods are usually low-cost and can be performed on-line. They can be divided into two groups: (a) the mechanism has more internal sensors than necessary; (b) a passive chain is added to the mechanism.

In [121], Yang used built-in sensors in the passive joints and the parameter errors were identified by the least-square algorithm. In [122], Hesselbach developed algorithms to determine the resolution of the required sensors to reach the desired accuracy. These algorithms can be easily adapted to any 6-DOF parallel mechanisms that consist of kinematic chains with 6-DOF. Chains must include at least one ball joint. The author designed an absolute angle measuring micro sensor system. This sensor is added into passive joints of parallel mechanisms. One of the objectives is therefore to obtain a robust compact sensor. The use of passive joint sensors usually simplifies the optimization procedure. In self-calibration, the measured passive joint angles and the calculated joint angles, by means of the model, can be compared. Hollerbach [123] used nine precision potentiometers to calibrate a 6-DOF parallel mechanism. The joint angle offsets and the joint angle gains were the identified kinematic parameters. These parameters related the raw analog input data from the potentiometers to the joint angles. The mechanism was placed into a number of poses, and the joint angles were read. The obtained potentiometer readings were converted to predicted joint angles and to predicted poses by solving the forward kinematics. In the following step, these values were compared with the theoretical joint angles obtained by means of the inverse kinematics.

##### Constrained calibration methods

Developments based on constraining the mobility do not require extra sensors [124,125]. In [124], Ryu analyzed a design which consisted of a link of fixed length with spherical ball joints in its ends. The measurement data is obtained by the internal measurement system of six actuators which measure the 5-DOF moving platform motion. This information is used in the calibration procedure without using any extra sensing device. The results show that the position and orientation error and the measurement noise and the link inaccuracy have the same order. Therefore, the calibration accuracy depends on the sensor accuracy and on the constraint link.

Constrained calibration methods decrease the number of DOF of the mechanism restricting the movement of the end-effector or the mobility of any joint. In these methods, the mechanism mobility is constrained during the calibration, thus some geometric parameters will remain constant in this process.

In [126], the mechanism was constrained in such a way that only platform rotations around a fixed point were allowed, and constraint equations were solved by means of the forward model. Chiu [127] limited the movement of a 6-UPS mechanism by adding a seventh leg, connected to the base through a universal joint linked to the end-effector. However, this design considerably restricts the working range and causes interferences between links. Besides, some parameter errors related to immobilize joints can occur unseen. In [128], Ren kept two attitude angles of the end-effector constant at different measurement configurations using a biaxial inclinometer.

These methods have lower costs than external calibration, but on the other hand they are more complex than self-calibration. Another problem is that not all the workspace is available due to constraints, and it is usually a less accurate method than external and self-calibration.

##### External calibration methods

However, in practice it is not easy to add extra redundant sensors or restrictions, so the most frequently used method is external calibration in which the necessary information is obtained by means of external devices such as theodolite [129], inclinometers [130,131], vision devices [117,132], the laser tracker [133,134] or the coordinate measuring machine [135–137].

Whitney [129] developed a forward calibration method. The method defined link lengths and joint sensor offsets as parameters. The theodolite measurements of tool position and the readings of the robot joint sensors were introduced in the model to perform the calibration procedure. The results show that the calibration model predicts theodolite readings with an error of 0.13 mm. Daney [117] developed a vision-based measurement method. Although the measurement data, for example the measured poses of the mechanism, are given by the sensor, it is necessary to consider the noise of this device. The mechanism poses were measured and sensors measured the six leg lengths for every pose.

Besnard [130] developed a method for the kinematic calibration of a 6-DOF parallel mechanism where the calibration model considered the error on the angle between the inclinometer axes. The two inclinometers were fixed to the platform. These sensors were used to measure the prismatic joint variables and the platform orientation. Each inclinometer measured its orientation from terrestrial horizontal, and both inclinometers had a null value when the platform was horizontal. The prismatic joint values and the inclinometer values were used to calibrate the geometric parameters for a number of configurations. The calibration procedure minimized the residual between the inclinometer measured values and the calculated values. The results show that inclinometers with a precision of 1e-3° and motorized joint sensors with a precision of 0.02 mm are necessary to obtain a position accuracy of about 0.4 mm. Rauf [131] developed a calibration method of parallel mechanism with partial pose measurements, by measuring the rotation of the end-effector along with its position. The device consists of a linear variable differential transformer and a biaxial inclinometer, to measure the position, and an optical encoder to measure the rotation. In [105], inclinometers were not used to measure precise values, rather to indicate if the values measured in one configuration were equal to the ones measured in a different configuration, thereby making the calibration method independent of the range of the measurements carried out and the positioning accuracy of the inclinometer. Although is not easy to obtain high accuracy and great workspace with conventional inclinometers, results obtained in calibration are usually satisfactory. The inclinometers were installed on the end-effector. The calibration was performed by keeping two attitude angles of the end-effector constant. The results show that the position and orientation accuracy, after calibration, can be 0.1 mm and 0.01°, when the inclinometer repeatability is 0.001°. And the precision of the leg length measurements is 0.002 mm. The results show that, before calibration, the errors were from 4 to 8 mm, and after calibration they were from −2 to 2 mm.

Renaud [132] developed a monocular high precision measuring device based on a vision sensor. A calibration target was placed in different positions and some images were taken with the vision sensor. The mathematical model obtained the pose of the target with respect to the vision sensor. The measuring device, based on a vision sensor, presents an accuracy on the order of 10 μm in position and 5e-4° in orientation; and, in [138], the author performed a calibration for a mechanism having linear actuators on the base. The forward model offers more stability, although it cannot converge if there are noise problems, and it is possible to perform the calibration by means of partial measurements of the end-effector pose.

In [133], Koseki used a laser tracking coordinate measuring machine. This device consisted of 4 laser stations and a wide-angle retro-reflector. When the laser beam was incident upon the retro-reflector, the reflected beams returned parallel to the incident beam. The retro-reflector had a lens whose focus coincided with its spherical surface and the surface mirrors incident beam. The interferometer measured the change in distance from the intersection of two axes of pan and tilt to the retro-reflector incrementally. Some advantages of this device are non-contact measurement, wide measuring range and ability to measure a high speed object. The results show that the accuracy obtained as the distance between measured position and calculated position presents an average error of 1.63 mm, before calibration, and 0.30 mm after calibration.

In [135] three methods were compared: a method using external measurement, a method using additional redundant sensors and a method using both. The author affirms that by using the implicit calibration, the basic system of equations may be obtained by using only the sensor information. The implicit calibration considers the basic system of equations obtained by the closed loop nature of parallel robots, and the system is specified by a function of the available data. The kinematic parameters can be identified by solving this system. The results show that the kinematic parameters were well identified as a function of the dimension of the redundant information on the state of the robot, and that the accuracy was higher for the method that used both: external measurements and additional redundant sensors.

In [139], Last classified the calibration techniques following the criteria of degree of automation and data-acquisition method. Thus, calibration techniques can be divided in four groups:

- Type 1: Non-autonomous methods by additional sensors from data acquisition, such as calibration by means of a laser tracker, camera systems or an extensible ball bar
- Type 2: Non-autonomous methods by kinematic constraints from data acquisition, such as calibration by contour tracking or by passive joint clamping
- Type 3: Autonomous methods by additional sensors from data acquisition, such as calibration by passive joint sensors or with actuation redundancy
- Type 4: Autonomous methods by kinematic constraints from data acquisition

##### How many sensors are necessary?

To assure that the number of equations is not smaller than the number of unknowns the minimum number of measurements, *m*, is given by Equation 3:

(3) m≥ρ+ζ

Defining *η* as the coefficients that relate the transducer signal (corresponding to the joint) to the real displacement of the joint and *a* as the coefficients in the kinematic model, *ρ* is the number of elements in the vector *η* and *ζ* is the number of elements in the vector *a* [94]. Besides, the positions chosen for data acquisition of the optimization process should guarantee the influence of all the parameters that are going to be identified, guaranteeing the generality of the parameters obtained for all the workspace [110]. In [140], Driels analyzed the optimum positions for data acquisition, and concluded that all the possible variation range of the joints in the mechanism should be covered. Nahvi [141] concluded that for performing a calibration, the number of joint sensors must be higher than the mobility of the mechanism. The author defined the noise amplification index and demonstrated that this index is an indicator of the amplification of the sensor noise and unmodeled errors. Moreover, the results show that the effects of sensor noise and unmodeled sources of error dominate the effect of length and other kinematic parameter variations of the mechanism. Merlet [13] explained that the number of constraint equations must be greater or equal than the number of unknowns. In practice the number of equations is usually greater, reducing the sensitivity of calibration to the uncertainty associated with measurements. This uncertainty is usually caused by measurement device noise. Thus, it is usual to develop an over-constrained system [142]. Huang [143] identified the parameter errors with an endpoint sensor and a dial indicator by measuring the flatness of a fictitious plane, the straightness and squareness of two orthogonal axes and the orientation error of the end-effector. These methods are usually based on data acquisition for fixed positions similar to work positions. Besides, it should be possible to generalize the parameter identification to positions different from those used for the identification process.

##### How many configurations must be measured?

The determination of the optimal number of configurations to the data acquisition, in order to perform a successful calibration, is still one of the unsolved problems, and in the specialized literature different criteria and opinions can be found. Therefore decisions are made without a specific methodology to obtain the configurations for a calibration process. According to Zhuang [144], the number of necessary configurations is given by n+1, n being the number of DOF of the mechanism. In [145], Borm defined the index of observability based on the non-zero singular values of the Jacobian matrix, and represented the data scatter. By maximizing this index, the errors of the parameters can be better observed. In [146], Sun related five observability indexes and analyzed how to minimize the variance of the parameters and minimize the uncertainty of the end-effector position. Agheli [147] showed that the boundaries of the workspace should be examined for the maximum observability errors. In [121], Yang illustrated the effect of the measurement noise and robot repeatability on the calibration results. If the measurement noise exists, more measured end-effector poses must be considered. The author simulated 100 end-effector poses (50 poses to calibrate the robot and 50 to verify the results), and he concluded that the quantified orientation and position deviations and the calibrated initial poses of the modules frames become stable when the number of poses used for calibration is greater than 20. The results show that the quantified orientation deviation becomes stable in 0.004 radians, and the quantified position deviation becomes stable in 0.09 mm. Bai [148] recommended that the calibration should consider more than 10 measured poses to improve the calibration accuracy. Moreover, in [105] Ren concluded that selecting an optimal set of configurations is more efficient in decreasing the influence of measurement noise. Also, increasing the number of measurement configurations will decrease the pose error but only in a limited way, as when the number of measurement configurations is increased over a certain amount the improvement is not clear but the runtime is increased considerably. The number of configurations needs to be adjusted to reach a balance between accuracy and efficiency. On the contrary, Horne [149] studied the effectiveness of five pose selection criteria: the geometric mean of the singular values, the inverse condition number, the minimum singular value, the noise amplification index and the inverse of the sum of the reciprocals of all of the singular values, and the results show that the pose selection criteria did not significantly improve the calibration process for the 4-DOF parallel mechanism studied, and moreover some of the results using the criteria are worse than those results where no criteria had been used.

(b) Optimization procedure

The calibration process can be solved by two ways [93]: (a) by multiplying the constraints by Lagrange multipliers and using a modified objective; (b) by solving the constraint equations and substituting into the objective functions.

In [93,114] authors applied Lagrange multipliers to perform the calibration. The model can be solved by means of Newton method.

The second method presents non-linear equations and the inverse matrix is not easy to obtain, however nowadays there are powerful computers that allow us to perform this type of procedure.

##### Define the objective function

The objective of the parameter identification or optimization is to search for the optimum values of all parameters included in the model that minimize the position error of the platform.

The objective function to minimize can be formulated in terms of a linear least-square problem. This function is usually defined as the quadratic difference of the error (obtained between the measured value of the end-effector position and the value computed by the kinematic model). The increment established for parameters must be defined for each iteration, and its value will depend on the optimization method chosen. In most of the cases numerical optimization techniques are used to minimize the end-effector error.

##### Objective function defined in terms of position and orientation of the end-effector (DKM)

The equation that relates joint variables with the final position of the end-effector by means of the forward method is given by the Equation 4:

(4) $$y=f\left(\theta_{i},p\right)$$

where *y* is the vector that expresses the position and orientation of the end-effector according to the Euler angles, *y=[x*, *y*, *z*, *α*, *β*, *γ]*, *θ_i_*, are the joint variables, with *i* from 1 to the number of DOF of the mechanism, and *p=[p*_1_, *p*_2_,*…*, *p_j_]^T^* is the vector of the model parameters. The number of parameters *j* will depend on the model chosen. Thus, it is possible to identify the geometric parameters from vector *p* by iterative optimization methods. These methods minimize the difference between the coordinates obtained by the model and the nominal values measured in the same position. These differences are denominated residues (see Equation 5):

(5) $$\phi =\sum_{i=1}^{n}{\left(y_{i}-f\left(\theta_{i},p\right)\right)}^{T}\left(y_{i}-f\left(\theta_{i},p\right)\right)$$

In this equation, *y_i_* is given by the vector of the nominal position and orientation values for the *n* configurations utilized in the parameter identification. In each configuration, the position and orientation of the end-effector will be obtained by means of the mechanism model given by *f*, for the joint variables *θ_i_*, corresponding to this configuration. This equation represents the objective function to minimize, whose value will be obtained as the sum of squares of the *n* poses, used in the parameter identification of the mechanism. A common way to express this equation is the one shown in Equation 6:

(6) $$\phi =\sum_{i=1}^{n}\left[{\left(x_{m_{i}}-x_{p_{i}}\right)}^{2}+{\left(y_{m_{i}}-y_{p_{i}}\right)}^{2}+{\left(z_{m_{\mathrm{ii}}}-z_{p_{i}}\right)}^{2}+{\left(\alpha_{m_{i}}-\alpha_{p_{i}}\right)}^{2}+{\left(\beta_{m_{i}}-\beta_{p_{i}}\right)}^{2}+{\left(\gamma_{m_{i}}-\gamma_{p_{i}}\right)}^{2}\right]$$

where the values with *m_i_* sub-index are the external measured values and the *p_i_* sub-index values are the computed values by means of the mathematical model, for the *n* identification poses. The optimum values will be given by the minimum of the objective function *ϕ*.

Traditionally, this equation has been widely used for open chain mechanisms [150], since in these systems is easier to obtain the forward kinematics, but in recent years it has also been widely used in parallel mechanisms such as in [93,95,104,105,136,143,147,148], where the position and orientation of the end-effector is measured and compared with the value given by applying the forward kinematic model, in function of model parameters and joint variables.

##### Objective function defined in terms of distances (IKM)

Another method to perform the calibration is by comparing the joint variables, which are given by the inverse kinematic model [115,117,123,138,141,151,152], by means of Equation 7:

(7) $$\phi =\sum_{i=1}^{n}{\left(q_{i}-g\left(X_{i},p\right)\right)}^{T}\left(q_{i}-g\left(X_{i},p\right)\right)$$

where *q_i_* are the joint variables, which are externally measured and *g(X_i_*, *p)* are the joint variables obtained by means of the inverse model, in function of the position and orientation of the platform and the model parameters.

Another widely used equation for obtaining the error is the representation as differences between the measured and the computed distances, *d_mi_* and *d_i_* respectively, obtained by the kinematic model, between two points, as it is shown in Equation 8:

(8) $$\phi =\sum_{i=1}^{n}{\left(d_{mi}-d_{i}\right)}^{2}$$

where the sub-index mi indicates the values externally measured, and the values with sub-index i correspond to the values obtained by the kinematic model.

Once the most suitable calibration model has been selected for the mechanism, and the objective function is defined, the next step will be to solve the system. These systems are non-linear, thus it is not possible to obtain an analytical solution to the parameter identification problem. Non-linear optimization iterative techniques are usually used to obtain the optimum parameters that minimize the error in the identification poses. For these systems, the most suitable resolution techniques are those based on least squares, specially used to adjust a parametric model to a set of data. A usual approach to the optimization problem consists of linearizing the equations of the model in an environment of the parameter to identify by means of a development in Taylor series. A suitable formulation to the optimization problem and a good approach to the function are achieved in a small interval in relation to its current value. In parallel kinematics it is usual to use developments in Taylor series for every parameter pi. This approach can be first or second order. The optimization problem can be solved through several methods:

(1) Gradient optimization methods

The simplest ones are those methods based on the gradient, also known as line search. These methods are usually used when the objective function to be minimized is approximated through a first-order Taylor series development. The following step will be to consider a stop criterion based on the convergence of the method or on small increments of parameters between iterations to obtain the set of parameters that minimizes the function.

(2) Least-square optimization methods

In the second group we can find those methods that consider second-order approaches in the Taylor series development. In this second term the Hessian matrix appears, with components that are the second derivatives of the objective function with respect to the vector of parameters. This matrix should be invertible. Problems derived from the singularity of this matrix in numerical optimization procedures must be solved by choosing a suitable mathematical model and a set of data for the optimization, or by employing optimization methods that avoid this singularity.

Optimization methods based on least-square are different from the previous ones when obtaining the direction of search and when defining the increment that parameters should have in this direction. One of the most frequently used least-square methods that take into account second order terms is the Gauss-Newton method. In this method the Hessian matrix should be positive or semi-defined positive, that is all its own values are positive or positives and zero, which will not always be produced for any mathematical model and for any set of values of the objective function. Moreover, convergence is not always guaranteed.

(3) Levenberg-Marquardt optimization method

The methods studied can present problems when processing the objective function, and are therefore not always suitable for the parameter identification process. The gradient method ensures that a local minimum of the function is found and usually requires more iterations to find it. It also needs the objective function to be continuous in every parameter, which not is always the case.

Numerical problems that appear in the two methods studied are solved by the algorithm developed by Levenberg and Marquardt [153,154]. The method adds a positive value *λ* to the Hessian main diagonal elements, obtaining a non-singular and therefore invertible matrix. The main problem of the Newton Gauss-Newton method with the processing of the second order components is therefore solved. To choose *λ*, a compromise between the speed of convergence of the method and the invertibility of the matrix must be reached in every iteration. This is why the Levenberg-Marquardt algorithm results in a combination of the two methods presented above. When parameters are far from the optimum solution, the value of *λ* increases and the algorithm behaves similarly to the gradient method; when the parameters approach their optimum value, the value of *λ* decreases, behaving in a similar way to the Gauss-Newton method in both search direction and parameter increment. This method is widely used in the calibration of parallel mechanisms by authors such as in [105,110,115, 123–126,130,132,135,143,151,155–158].

In the specialized literature we can find other alternatives to the Gauss-Newton method problem. For example, the singular value decomposition (SVD) [114,116,147,148,156,159].

Another alternative is the use of the decomposition QR for parameter identification [125]. In [160] an algorithm allows us to introduce range limits of the joints in a configuration selection process and avoids the problem of local minimum, although it is a high-cost computational method. In [161,162] the problem is solved by means of genetic algorithms. Yu applies the inverse kinematics model and improves the accuracy in the parallel robot position by means of an artificial neural network. In [163], Stan performs the optimization of a 2-DOF parallel robot using genetic algorithms. The author considers transmission quality index, manipulability, stiffness and workspace. In [164] Liu obtains residual measures through inverse kinematics and develops a calibration method using genetic algorithms.

Techniques based on least-square usually present lower computational cost, providing we consider as an initial value a solution close to the optimum solution for the set of parameters. However, in methods based on genetic and neural networks algorithms this premise is not usually so significant. These algorithms are usually used for parameter optimization and identification when it is not known whether the initial values are close to the optimum solution. Furthermore, the combinatorial nature of these methods is purely stochastic, which avoids problems in the definition of the search direction in traditional least-square methods.

(c) Evaluation of the identified parameters

The evaluation of the identified parameters consists of evaluating the mechanism behaviour with the set of the identified parameters obtained in the previous step. This procedure is performed in configurations different from those utilized in the optimization process. This phase must evaluate the degree of compliance of the error values obtained in other positions of the workspace. In [133], Koseki utilized a laser tracking coordinate measuring system to evaluate the accuracy of a parallel mechanism. Cheng [165] analyzed the relationship between original errors and position-stance error of a moving platform by means of the complete differential-coefficient theory and evaluates the error model. The conclusion is that improving manufacturing and assembly techniques allows us to reduce the moving platform error, and that a small change in position-stance error in different kinematic positions proves that the error-compensation of software can considerably improve the precision of parallel mechanisms.

(d) Correction model

To end the calibration process, a correction model can be obtained to improve the accuracy of the mechanism. Huang [166] performed the calibration of a parallel mechanism and compensated geometrical and position errors in *x* and *y* coordinates. Gong [111] identifies non-geometric errors and developed a method to compensate these errors with a laser tracker by means of the inverse calibration model. An extensive guide of error compensation methods can be found in [167]. In this paper, Oiwa explained in detail how to compensate joints errors, link length, forces in a measurement loop and frame deformation using a coordinate measuring machine. This method considers thermal effect and external forces. The results show that the deflection of measured Z-coordinates is not completely eliminated with the compensation system, but compensation using displacement sensors built in spherical joints improves moving accuracy of parallel kinematic mechanisms when the mechanism moves in a large working space. The thermal and elastic deformations of the limb can be compensated by connecting the scale unit with the joints through low expansion material rods. Bringing the rod end into contact with the ball of the spherical joint enables the scale unit to measure the joint error and the limb deformations. And, the measured position and the orientation of the base platform compensate the thermal and elastic deformations of the frame.

## 5. Conclusions

This paper presents an overview of the solutions developed on kinematic and calibration models of parallel mechanisms and the influence of sensors in the mechanism accuracy in recent years. The most relevant classifications to obtain and solve kinematic models and to identify geometric and non-geometric parameters in the calibration of parallel robots are presented. And the advantages and disadvantages of these methods, applications of parallel mechanisms as sensors, new trends and the identification of unsolved problems are discussed. This overview is intended to be a guide for researchers working on parallel mechanisms, in the design, modelling and calibration of these systems. In the document, some common questions are answered and the most up-to-date research carried out is summarized. The document describes the different phases required to perform a calibration process, putting special emphasis on the fact that the first decision made, the calibration method, will determine the number and type of necessary sensors, internal or external, from data acquisition, the required configurations and the calibration model. The model will determine the nature and number of necessary parameters. There are different methods to perform the calibration process, and the choice of one or the other must consider the characteristics of the mechanism. Methods based on computational intelligence are able to scan a vast solution set and are not as sensitive to bad initial values as classical methods. These methods can be suitable for those systems where it is very complex or impossible to obtain the model by means of classical methods. On the contrary, in those devices in which a careful kinematic analysis is necessary to obtain complete performance knowledge, classical methods are more suitable. Depending on the type and number of selected sensors, the cost function will be formulated. This function is established in terms of position and orientation of the moving platform (DKM) or in terms of distances (IKM). And, finally, the evaluation of the results and the correction model will allow us to improve the accuracy of the system.

## Figures and Tables

**Table 1.** Joint symbols.

**Symbol Joint**	**Type of joint**
**U**	Universal joint
**P**	Prismatic joint
**R**	Revolute joint
**S**	Spherical joint
**Pa**	Parallelogram joint
